# Chlamydia trachomatis Plasmid Gene Protein 3 Is Essential for the Establishment of Persistent Infection and Associated Immunopathology

**DOI:** 10.1128/mBio.01902-20

**Published:** 2020-08-18

**Authors:** Chunfu Yang, Laszlo Kari, Lei Lei, John H. Carlson, Li Ma, Claire E. Couch, William M. Whitmire, Kevin Bock, Ian Moore, Christine Bonner, Grant McClarty, Harlan D. Caldwell

**Affiliations:** a Laboratory of Clinical Immunology and Microbiology, National Institute of Allergy and Infectious Diseases, National Institutes of Health, Bethesda, Maryland, USA; b Laboratory of Bacteriology, Rocky Mountain Laboratory, National Institute of Allergy and Infectious Diseases, National Institutes of Health, Hamilton, Montana, USA; c Comparative Medicine Branch, National Institutes of Allergy and Infectious Diseases, National Institutes of Health, Bethesda, Maryland, USA; d National Microbiology Laboratory, Public Health Agency of Canada, Winnipeg, Manitoba, Canada; e Department of Medical Microbiology, University of Manitoba, Winnipeg, Manitoba, Canada; University of Nebraska Medical Center

**Keywords:** *Chlamydia*, plasmid, persistent infection, antimicrobial peptides

## Abstract

Chlamydia trachomatis is an obligate intracellular bacterial pathogen that causes blinding trachoma and sexually transmitted disease afflicting hundreds of millions of people globally. A fundamental but poorly understood pathophysiological characteristic of chlamydial infection is the propensity to cause persistent infection that drives damaging inflammatory disease. The chlamydial plasmid is a virulence factor, but its role in the pathogenesis of persistent infection capable of driving immunopathology is unknown. Here, we show by using mouse and nonhuman primate infection models that the secreted plasmid gene protein 3 (Pgp3) is essential for establishing persistent infection. Ppg3-dependent persistent genital tract infection resulted in a severe endometritis caused by an intense infiltration of endometrial submucosal macrophages. Pgp3 released from the cytosol of lysed infected oviduct epithelial cells, not organism outer membrane-associated Pgp3, inhibited the chlamydial killing activity of antimicrobial peptides. Genetic Pgp3 rescue experiments in cathelin-related antimicrobial peptide (CRAMP)-deficient mice showed Pgp3-targeted antimicrobial peptides to subvert innate immunity as a pathogenic strategy to establish persistent infection. These findings provide important advances in understanding the role of Pgp3 in the pathogenesis of persistent chlamydial infection and associated immunopathology.

## Abstract (precis)

Chlamydia trachomatis can cause persistent infection that drives damaging inflammatory responses resulting in infertility and blindness. Little is known about chlamydial genes that cause persistence or factors that drive damaging pathology. In this work, we show that the C. trachomatis plasmid protein gene 3 (Pgp3) is the essential virulence factor for establishing persistent female genital tract infection and provide supportive evidence that Pgp3 functions similarly in a nonhuman primate trachoma model. We further show that persistent Ppg3-dependent infection drives damaging immunopathology. These results are important advances in understanding the pathophysiology of chlamydial persistence.

## INTRODUCTION

Chlamydia trachomatis (Ct) is an obligate intracellular bacterial pathogen distinguished by a unique biphasic developmental growth cycle (1) that causes blinding trachoma and sexually transmitted infection (STI). Trachoma is a disease of developing countries and responsible for the blindness or visual impairment of approximately 1.9 million people (2). Ct infection is a leading cause of STI worldwide, and in the United States alone, there are 1.7 million reported cases of infection per year (3). A sequela of female infection is pelvic inflammatory disease (PID)-associated pathology that may result in tubal factor infertility and ectopic pregnancy (4). The pathophysiology of PID is not fully understood, but reinfection or persistent infection that drives damaging inflammatory immunopathology is thought to be important (5–9), which is corroborated by animal models of reinfection (10, 11); conversely, animal models of persistent Ct infection that drive damaging pathology have not been reported.

Ct isolates share a 7.5-kb virulence-associated plasmid (12) carrying eight open reading frames (ORF) encoding proteins designated plasmid gene proteins 1 to 8 (Pgp1 to Pgp8). Pgp1, Pgp2, Pgp6, and Pgp8 are important for plasmid maintenance (13, 14). Pgp4 is a master positive regulator of plasmid-encoded Pgp3 and the chromosomal genes, including GlgA, CT049-CT050, and CT142-CT144 (13), whereas Pgp5 is a negative regulator of Pgp4-regulated genes (15). Pgp7 is a homologue of Pgp8 and shows homology to integrases (13). The plasmid encodes two small antisense RNAs (sRNAs) (16) implicated in plasmid maintenance (14). Plasmid-deficient strains (17–21) and Pgp3 and Pgp4 null mutant strains (19, 22) exhibit attenuated infection characteristics in nonhuman primate and murine infection models. Plasmid-deficient organisms revealed a significant reduction in shedding duration in the ocular trachoma model (17), leading to the hypothesis that the plasmid plays a role in the establishment of persistent infection (23). Importantly, while plasmid-free C. trachomatis clinical isolates exist, they are exceedingly rare (24), supporting a critical role for the plasmid in the host-pathogen transmission cycle. The presence of plasmid is also associated with increased risk of PID (25).

Antimicrobial peptides (AMPs) are the first line of innate defense against invading microorganisms of skin and mucosal surfaces. The female reproductive tract expresses multiple AMPs, including defensins, cathelicidin, S100 proteins, C-type lectins, and iron metabolism proteins (26). Defensin and cathelicidin (LL-37) are known to kill chlamydial organisms by lysing infectious elementary bodies (EBs) (27–29). Bacterial pathogens have different strategies to counteract AMPs to promote colonization and infection (26). Pgp3 is secreted from the inclusion bodies into the host cytosol (30) and neutralizes the antichlamydial activity of cathelicidin LL-37 in *in vitro* cell culture assays (29). However, a role for AMPs in innate host defense in models of chlamydial *in vivo* infection have not been reported. Here, we show that Pgp3 is essential for the establishment of persistent infection that drives damaging inflammatory disease. We describe Pgp3 inhibition of AMP host defense as a pathogenic mechanism to subvert innate immunity and facilitate the establishment of persistent infection. Mechanistically, we show that secreted Pgp3, not Pgp3 associated with infectious EBs, inhibits the antichlamydial activity of AMPs.

## RESULTS

### The C. trachomatis plasmid is essential for establishing persistent infection in the female mouse GT.

**FIG 1:**
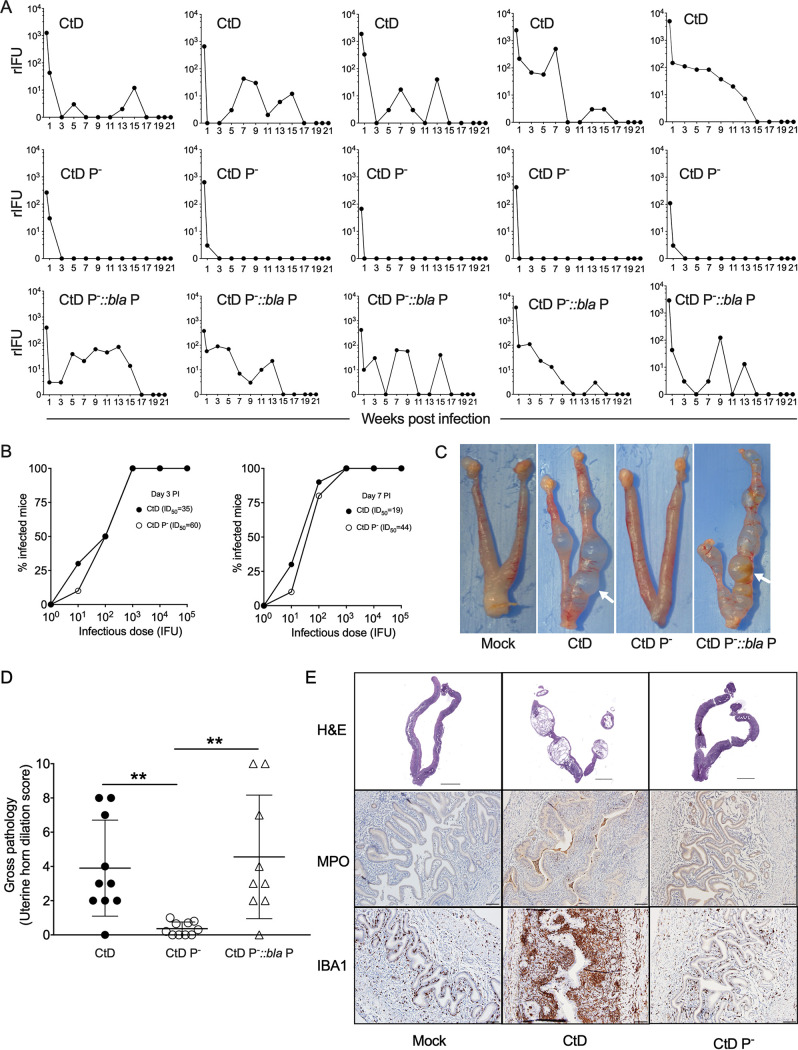
The chlamydial plasmid is essential for establishing persistent infection. (A) Chlamydial burden and shedding kinetics in the female mouse genital tract. C57BL/6 mice (*n* = 5) were infected transcervically with 3 μl SPG containing 1 × 10^5^ IFU of CtD, CtD P^−^, and CtD pBRDUW3 (CtD P^−^::*bla* P). Recoverable inclusion-forming units (rIFU) in vaginal swabs were assayed on the indicated days p.i. The rIFU of CtD- and CtD P^−^-infected mice were significantly different on day 3 (*P* = 0.0079), week 1 (*P* = 0.0437), week 2 (*P* = 0.0079), week 3 (*P* = 0.0476), and week 6 (*P* = 0.0079) p.i. The total infectious burdens of CtD- and CtD P^−^-infected mice were significantly different (*P* = 0.0079). The rIFU of CtD pBRDUW- and CtD P^−^-infected mice were significantly different at week 1 (*P* = 0.0475), week 2 (*P* = 0.0420), and week 3 (*P* = 0.0079) p.i. The total infectious burdens of CtD pBRDUW3- and CtD P^−^-infected mice were significantly different (*P* = 0.0317). The Mann-Whitney test was used. (B) ID_50_ of CtD and CtD P^−^ in the female mouse genital tract. C57BL/6 mice (*n* = 10) were infected transcervically with CtD or CtD P^−^. Recoverable IFU in vaginal swabs were assayed at day 3 and 7 p.i. The ID_50_ of the CtD and CtD P^−^ strains at day 3 and day 7 are calculated by logit analysis. (C) Gross pathology of the genital tracts of mock-, CtD-, CtD P^−^-, and CtD pBRDUW3-infected mice at day 60 p.i. CtD- and CtD pBRDUW3-infected mice exhibit severe uterine horn dilation characterized by predominant protrusions (white arrows) extending from the uterine horns. (D) Quantification of uterine horn gross pathology. The clinical disease severity of CtD-, CtD P^−^-, and CtD pBRDUW3-infected mice was scored (0 to 10) by counting the total number of protrusions on both uterine horns from individual mice. (**, *P* < 0.01 by the Mann-Whitney test). (E) Histopathology and immunohistochemistry of genital tract tissue from CtD- or CtD P^−^-infected C57BL/6 mice (*n* = 3). Tissue was stained with hematoxylin and eosin (H&E), rabbit polyclonal antimyeloperoxidase (anti-MPO) or anti-IBA1 antibodies to detect neutrophils and macrophages, respectively. Histopathology in CtD-infected mice was characterized by severe endometrial gland dilation, stromal atrophy, and leukocyte infiltration comprised primarily of luminal neutrophils and subluminal macrophages. These histological changes and leukocyte staining patterns were minimal to absent in the tissues of CtD P^−^-infected mice.

Ct infection causes persistent genital tract (GT) infection in humans that can persist for years (6–8, 31). The Ct virulence factors required for the establishment of persistent infection are unknown. We therefore sought to investigate a potential role for the plasmid in the establishment of persistent infection using a female mouse genital tract infection model and a human urogenital challenge strain. C57BL/6 mice were infected transcervically with Ct D/UW-3/Cx plasmid-positive (CtD), plasmid-negative (CtD P^−^), and plasmid-complemented (CtD pBRDUW3) strains (Fig. 1A). We found CtD infection persisted for a period of 13 to 15 weeks. Persistently CtD-infected mice exhibited alternating periods of culture negativity and positivity that spanned the entire 21-week culture period. In contrast, CtD P^−^-infected mice exhibited a marked difference in infection kinetics. Mice were culture positive only during the first 3 weeks and remained negative for the remaining culture period. The genetically complemented CtD pBRDUW3 strain displayed infection kinetics similar to those of CtD, demonstrating that the persistent phenotype is plasmid dependent.

To determine whether plasmid-mediated persistent infection was the result of differences in colonization efficiency, 50% infectious doses (ID_50_) of CtD and CtD P^−^ strains were determined. CtD and CtD P^−^ strains inoculated transcervically had very similar ID_50_ values at days 3 and 7 postinfection (p.i.) (Fig. 1B), supporting the conclusion that the plasmid-dependent persistence phenotype is not due to differences in GT colonization efficiency. In contrast to our finding, two previous studies showed that Ct P^−^ strains had reduced colonization compared to their P^+^ counterparts in the mouse GT infection model (18, 20). A possible explanation for this discrepancy is that we employed transcervical inoculation, while intravaginal inoculation was performed in the previous studies. In support of this, Gondek et al. (32) showed that Ct transcervical inoculation results in more efficient colonization than intravaginal inoculation.

In women, persistent GT infection is associated with chronic inflammatory disease of unknown pathophysiology but has been linked to innate immune mechanisms (33). Genital tract tissues of mice infected with CtD, CtD P^−^, and CtD pBRDUW3 were examined histologically and immunochemically. CtD-infected mice developed severe uterine horn dilation that was restricted to uterine horns, with no evidence of upper genital tract disease resulting in hydrosalpinx (Fig. 1C and D). These pathological findings are similar to those reported by other investigators using urogenital Ct strains in mice (34, 35). In contrast, CtD P^−^-infected mice exhibited no, or only minimal, uterine horn dilation (Fig. 1C and D). Mice infected with the plasmid-complemented strain CtD pBRDUW3 restored uterine horn pathology. Histopathology and immunohistochemistry in CtD-infected mice were characterized by severe endometritis resulting in endometrial gland dilation, stromal atrophy, and leukocyte infiltration comprised primarily of luminal neutrophils and submucosal macrophages (Fig. 1E). These histological changes and leukocyte staining patterns were minimal to absent in the tissues of CtD P^−^-infected mice (Fig. 1E). Collectively, the results clearly demonstrate a role for the Ct plasmid in the establishment of persistent genital tract infection resulting in damaging macrophage-associated immunopathology.

### Pgp3 is required for establishing persistent infection.

**FIG 2:**
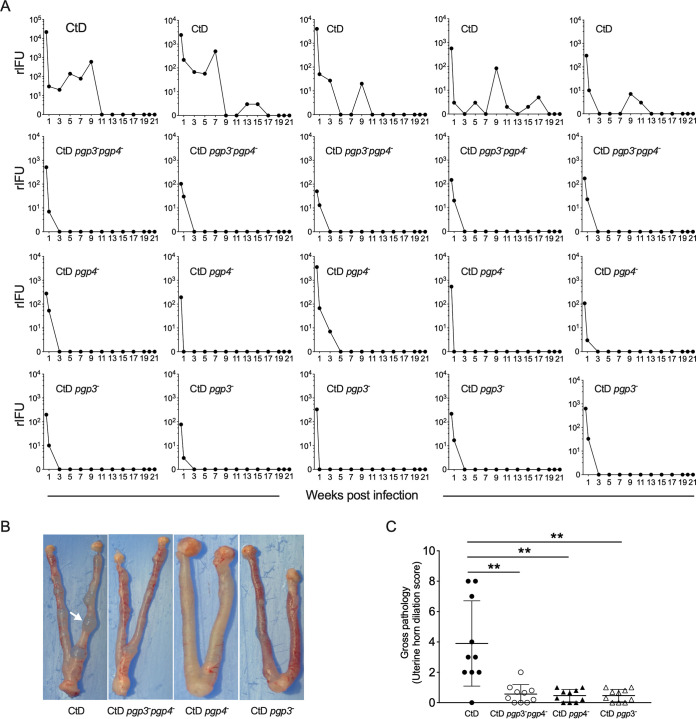
Pgp3 is required for persistent infection in the female mouse genital tract. (A) Chlamydial burden and shedding kinetics in the female mouse genital tract. C57BL/6 mice (*n* = 5) were infected transcervically with 3 μl SPG containing 1 × 10^5^ IFU of CtD, CtD *pgp3*^–^ *pgp4*^–^, CtD *pgp4*^–^, or CtD *pgp3*^–^. rIFU in vaginal swabs were assayed at the indicated time points p.i. The rIFU of mice infected with CtD and CtD *pgp3*^–^ (*P* = 0.0278), CtD and CtD *pgp4*^–^ (*P* = 0.047678), and CtD and CtD *pgp3*^–^ *pgp4*^–^ (*P* = 0.0080) were significantly different on day 3. The total infectious burdens in mice infected with CtD and CtD *pgp3*^–^ (*P* = 0.0214), CtD and CtD *pgp4*^–^ (*P* = 0.0263), and CtD and CtD *pgp3*^–^ *pgp4*^–^ (*P* = 0.00262) were significantly different. The Mann-Whitney test was used. (B) Gross pathology of the genital tracts of mice infected with CtD, CtD *pgp3*^–^ *pgp4*^–^, CtD *pgp4*^–^, and CtD *pgp3*^–^ on day 60 p.i. CtD-infected mice exhibit severe uterine horn dilation characterized by predominant protrusions (white arrow) extending from the uterine horns. (C) Quantification of uterine horn gross pathology. Clinical disease severity of CtD, CtD *pgp3*^–^ *pgp4*^–^, CtD *pgp4*^–^, and CtD *pgp3*^–^-infected mice was scored (0 to 10) by counting the total number of protrusions on both uterine horns from individual mice. (**, *P* < 0.01 by the Mann-Whitney test).

Pgp3 and Pgp4 are key genes in plasmid-mediated pathogenesis (13, 19). Pgp4 is a transcriptional regulator of Pgp3 and various chromosomal virulence genes (13), but the role these genes play in persistent infection is unknown. We therefore made CtD *pgp3^–^*, CtD *pgp4^–^*, and CtD *pgp3^–^ pgp4^–^* mutant strains (see Fig. S1 in the supplemental material) and tested their infectivity in the mouse GT. Female C57BL/6 mice were inoculated transcervically with the CtD, CtD *pgp3^–^ pgp4^–^*, CtD *pgp4^–^*, and CtD *pgp3^–^* strains. As expected, CtD-infected mice exhibited similar profiles in infection burden and duration. However, like the CtD P^−^ strain, the infection kinetics of mice infected with CtD *pgp3^–^ pgp4^–^*, CtD *pgp4^–^*, or CtD *pgp3^–^* were highly attenuated, generating a transient self-limiting infection (Fig. 2A). Because infection with the CtD *pgp3^–^* strain produced an infection profile nearly identical to that of the CtD P^−^, CtD *pgp3^–^ pgp4^–^*, or CtD *pgp4^–^* strain, we excluded Pgp4-regulated chromosomal genes as virulence factors in causing persistent urogenital tract infection. As with infection kinetics, mice infected with the CtD *pgp3^–^*, CtD *pgp4^–^*, and CtD *pgp3^–^ pgp4^–^* strains showed no or minimal uterine horn dilation (Fig. 2B and C). We therefore conclude that Pgp3 is the key plasmid virulence factor required for establishing persistent infection capable of causing immunopathology.

10.1128/mBio.01902-20.1 FIG S1 Phenotypic characterization of CtD, CtD P^−^, and transformants. Western blotting showed that Pgp3, CT143, and CT144 were not expressed in the CtD P^−^, CtD *pgp3*^–^ *pgp4*^–^, and CtD *pgp3*^–^ strains. Minimal expression of Pgp3 was found in the CtD *pgp4*^–^ strain, indicating a basal level of *pgp3* gene expression. The purpose of including CT143 and CT144 is to show that expression of these proteins is regulated by Pgp4. Download FIG S1, TIF file, 0.4 MB. Copyright © 2020 Yang et al. 2020 Yang et al. This content is distributed under the terms of the Creative Commons Attribution 4.0 International license.

### Genetic rescue of CtD *pgp3^–^* infectivity in female CRAMP knockout (KO) mice.

**FIG 3:**
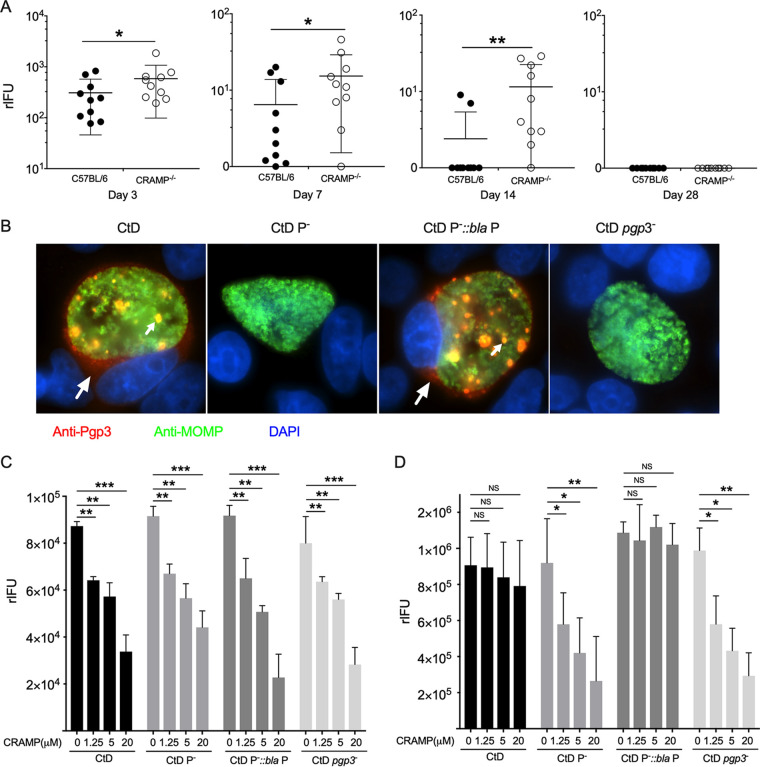
Genetic rescue of CtD *pgp3*^–^ infectivity in female CRAMP KO mice and a molecular mechanism of Pgp3-mediated inhibition of AMP activity. (A) Genetic rescue of CtD *pgp3*^–^ infectivity in CRAMP gene-deficient mice. Ten female C57BL/6 or CRAMP KO mice were infected transcervically with 1 × 10^5^ IFU of CtD *pgp3*^–^ organisms, and infectious loads (rIFU) present in cervicovaginal swabs were determined at days 3 to 28 p.i. The *P* values were calculated with a Mann-Whitney test. (B) Immunofluorescence of HeLa229 cells infected with CtD, CtD P^−^, CtD pBRDUW3, and CtD *pgp3*^–^ at 40 h p.i. stained with anti-Pgp3 (red), anti-MOMP (green), and DAPI (blue). Arrows denote inclusion body and secreted cytosolic Pgp3 in CtD- and CtD pBRDUW3-infected cells. (C and D) Secreted extracellular Pgp3 inhibits AMP killing of chlamydiae. (C) CtD, CtD P^−^, CtD pBRDUW3, and CtD *pgp3*^–^ organisms were incubated with different concentrations of CRAMP for 1.5 h at room temperature, and infectivity was assayed by titration of IFU on McCoy cells. CRAMP inhibited infectivity of all strains in a dose-dependent manner but, importantly, was not Pgp3 dependent. The *P* values were calculated with Student’s *t* tests. (D) Supernatants from osmotically lysed cells infected with CtD, CtD P^−^, CtD pBRDUW3, and CtD *pgp3*^–^ at 40 h pi were incubated with different concentrations of CRAMP for 1.5 h at room temperature, and infectivity was assayed by titration of rIFU on McCoy cells. The infectivity of CtD and CtD pBRDUW3 was not significantly reduced at any concentration. In marked contrast, the infectivity of CtD P^−^ and CtD *pgp3*^–^ was significantly reduced in a dose-dependent manner following CRAMP treatment. Statistical significance by Student’s *t* test is indicated as follows: NS, not significant; *, *P* < 0.05; **, *P* < 0.01; ***, *P* < 0.001.

Our findings demonstrate that Pgp3 is essential for establishing persistent infection and led us to design experiments to define the responsible mechanism. Several studies have shown that defensins and cathelicidin possess potent antimicrobial efficacy against chlamydiae (27–29). Moreover, recombinant Pgp3 (rPgp3) binds to cathelicidin LL-37 and neutralizes its antichlamydial activity *in vitro* (29). A role for Pgp3 counteracting AMPs *in vivo* has not been shown. To determine whether Pgp3 can protect Ct from AMP killing *in vivo*, we infected cathelin-related antimicrobial peptide (CRAMP)-deficient mice with the CtD and CtD *pgp3^–^* strains. Cathelin-related antimicrobial peptide is the mouse counterpart to human cathelicidin LL37 (36). The course of infection was monitored by culturing vaginal swabs for Ct. We found that the burden of CtD *pgp3^–^*-infected CRAMP^−/−^ mice was significantly higher than that of CtD *pgp3^–^*-infected C57BL/6 mice at days 3, 7, and 14 p.i. (Fig. 3A). These results support a role for CRAMP in the clearance of Ct infection and for Ppg3 in the neutralization of CRAMP *in vivo*. Notably, both CtD *pgp3^–^*-infected CRAMP^−/−^ and C57BL/6 mice resolved infection by day 28 p.i. This result is not unexpected, given that there are multiple AMPs with overlapping and synergistic antimicrobial properties in the mouse genital tract (26).

### Secreted Pgp3 inhibits CRAMP.

We next investigate a molecular mechanism for how Pgp3 subverts CRAMP antichlamydial activity. rPgp3 binds to cathelicidin LL-37 and neutralizes its antichlamydial activity *in vitro* (29). Pgp3 is found in the chlamydial outer membrane complex (37) and secreted into the host cytosol (30). We performed indirect immunofluorescence assay (IFA) staining with anti-Pgp3 antibodies in McCoy cells infected with CtD, CtD P^−^, CtD pBRDUW3, and CtD *pgp3^–^*, which confirmed Pgp3 expression and host cell cytosolic localization (Fig. 3B). We envisioned two potential ways by which Pgp3 could shield Ct from AMP function. (i) Pgp3 associated with EB outer membrane complex binds CRAMP, thus directly blocking antichlamydial activity. (ii) Secreted Pgp3, released into the extracellular environment following host cell lysis or inclusion extrusion binds CRAMP, thus inhibiting its antichlamydial activity. To investigate the first possibility, CtD, CtD P^−^, CtD pBRDUW3, and CtD *pgp3^–^* organisms were incubated with different concentrations of CRAMP, and infectivity was assayed (Fig. 3C). We found that CRAMP inhibited the infectivity of all strains in a dose-dependent manner. Importantly, however, CRAMP inhibition was Pgp3 independent, showing that EB-associated Pgp3 fails to provide protection against CRAMP.

We next asked whether cytosolic Pgp3 released into the lumen of the infected genital tract following infected cell lysis could bind and inactivate CRAMP. Cells infected with CtD, CtD P^−^, CtD pBRDUW3, and CtD *pgp3^–^* were osmotically lysed at 44 h p.i., and cell lysates were incubated with different concentrations of CRAMP (Fig. 3D). The infectivities of CtD and CtD pBRDUW3 were not affected following CRAMP treatment. In contrast, the infectivities of CtD P^−^ and CtD *pgp3^–^* were significantly reduced in a dose-dependent manner following treatment. The ability of extracellular Pgp3 to neutralize CRAMP is consistent with the ability of rPgp3 to neutralize CRAMP antichlamydial activity *in vitro* (29). Collectively, our findings indicate that secreted Pgp3 released from lysed cells, not Pgp3 associated with EBs, functions to inhibit the antichlamydial activity of CRAMP, thereby facilitating the establishment of persistent infection.

### A C. trachomatis Pgp3 null ocular strain does not produce persistent infection in nonhuman primates.

**FIG 4:**
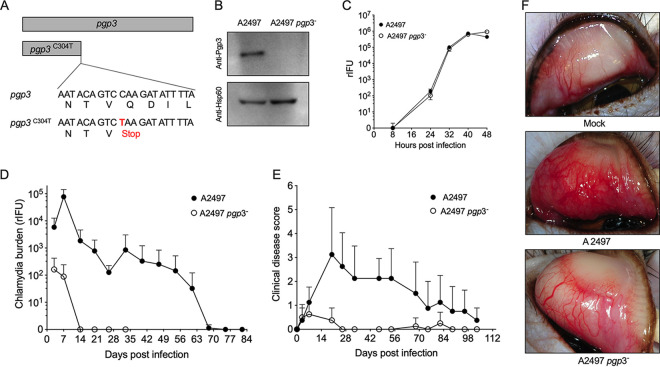
Pgp3 is required for establishing persistent ocular infection in nonhuman primates. (A) Schematic depiction of the predicted ORF of Pgp3 in C. trachomatis ocular strain A2497. The *pgp3* mutant contains an inactivating stop codon at C304T. (B) Western blotting showed that Pgp3 was not expressed in the A2497 *pgp3*^–^ strain. (C) A2497 and A2497 *pgp3*^–^ strains exhibit similar growth kinetics in McCoy cells. One-step growth curves of McCoy cells were determined. McCoy cells were infected at an MOI of 0.5. Cells were harvested at different times postinfection, and the number of rIFU was determined. Mean and standard deviation are shown (*n* = 3). (D) Ocular infection of macaques (*n* = 4) infected with the A2497 and A2497 *pgp3*^–^ strains. (E) Ocular clinical disease scores of macaques (*n* = 4) infected with the A2497 and A2497 *pgp3*^–^ strains. (F) Representative images of macaque eyes infected with A2497 and A2497 *pgp3*^–^ strains.

Persistent infection is thought to play an important role in the pathogenesis of blinding trachoma (7); therefore, we asked whether Pgp3 might play a similar role in the establishment of persistent infection in a trachoma nonhuman primate ocular infection model. Nonhuman primates are the only animal model susceptible to ocular infection and disease caused by trachoma strains. We used chemical mutagenesis and reverse genetics to isolate a *pgp3* null isolate from the trachoma 2497 strain (38). The A2497 *pgp3^–^* strain has an inactivating stop codon at C304T (Fig. 4A) that was confirmed by Western blotting (Fig. 4B). The A2497 wild-type and *pgp3^–^* strains exhibited similar *in vitro* growth characteristics (Fig. 4C). In contrast, the infection profiles of these strains following ocular challenge of nonhuman primates were strikingly different. Infection with wild-type A2497 organisms persisted for up to 70 days p.i. and produced moderate to severe ocular inflammatory pathology. In contrast, infections with the A2497 *pgp3^–^* strain did not persist but were spontaneously cleared at day 14 p.i. and resulted in minimal ocular pathology (Fig. 4D to F). Collectively, our findings showing similar results using Pgp3^−^ organisms from ocular and genital Ct strains in two independent infection models provides conclusive evidence that Pgp3 is a central and key virulence factor in the establishment of persistent chlamydial infection.

## DISCUSSION

The importance of persistent Ct infections that drive chronic damaging inflammatory responses has been proposed for blinding trachoma and PID (6–8), but to date, direct evidence to support this hypothesis is lacking. Here, we showed that Ct causes persistent infection of the female mouse genital tract that triggers severe endometritis characterized by the infiltration of submucosal macrophages. We further showed that the Ct plasmid is required for establishment of persistent genital tract infection and that Pgp3 is the essential virulence factor. Mechanistically Ppg3’s ability to bind and inactivate AMPs is the function essential for the development of persistence. Whether Pgp3 might have additional pathogenic mechanisms for avoiding host defenses during persistent infection remains to be determined. Importantly, we corroborated the importance of Pgp3 as a virulence factor in persistent infection using a trachoma biovar and nonhuman primate ocular infection model. Collectively, our findings provide experimental evidence supporting a role and biological mechanism for Pgp3 in the pathogenesis of persistent chlamydial infection.

Our results indicate that secreted Pgp3 released from lysed cells, not Pgp3 associated with EBs, functions to subvert the antichlamydial activity of AMP. The middle portion of Pgp3 is a prominent negatively charged region located in a triple-helical coiled coil adjacent to the C-terminal domain (39). This domain is postulated to be critical for neutralization of LL37 (29). Like LL37, many other AMPs are cationic amphipathic peptides; thus, Pgp3 might possess a potentially broad biological effect in the inhibition of many positively charged AMPs through electrostatic interaction. Compared to wild-type animals, mice with a single AMP deficiency showed a significant decrease in CtD *pgp3*^–^ burden. Considering that many cationic AMPs with redundant antimicrobial properties are likely present in the female mouse GT, it is logical that Ppg3 deficiency would exhibit a more dramatic persistence phenotype in mice lacking multiple AMPs, but unfortunately, such mice are not available.

Pgp4 regulates the expression of Pgp3 and multiple chromosomal genes (13). In this study, we found that persistent infection was Pgp3 dependent. We observed that the persistent phenotype of the CtD *pgp4*^–^ strain was similar to that of CtD *pgp3*^–^. These findings imply that Pgp4-regulated chromosomal genes do not play a direct pathogenic role in the murine persistent-infection model. This raises the question of the function of these genes in the pathogenesis of chlamydial infection. Might they play an indirect role in assisting the function of Pgp3 by facilitating its secretion into the host cytosol? Chlamydial exit from host cells occurs by inclusion body extrusion (40) and plasmid-dependent host cell lysis (41). Although mechanistically different, both release mechanisms ensure that secreted cytosolic Pgp3 is exported extracellularly to function as a virulence factor to target inactivation of luminal AMPs.

Innate immunity is implicated in the immunopathogenesis of trachoma and PID (33). Transcriptional profiling of trachoma conjunctival samples showed transcriptional networks connected to the innate immune response (42, 43). Biomarker studies of women with chlamydial endometritis found increased expression levels of myeloid mediators of inflammation (44). We found that Pgp3-mediated persistent infection caused chronic macrophage immunopathology in the mouse model. Collectively, these findings and the findings reported here support the possibility that macrophages may be a fundamental cause of immunopathogenesis in human chlamydial diseases. These findings raise the question of what chlamydial virulence factors and pathogenic mechanisms drive macrophage-mediated inflammatory disease?

## MATERIALS AND METHODS

### Chlamydiae.

A low-passage-number C. trachomatis D/UW-3/Cx strain (CtD strain) was plaque cloned and whole genome sequenced. The CtD strain harbors an intact CT135 gene that encodes a known virulence factor in the murine model (45). The plasmid-free strain (CtD P^−^) was derived by novobiocin treatment as previously described (46). The shuttle vector pBRDUW3 was used as the template to make the nonsense mutants CtD *pgp*3T212A, CtD *pgp*4A37T, and CtD *pgp*3T212A *pgp*4A37T as previously described (14). The CtD P^−^ strain was transformed with each expression construct to obtain the transformants CtD pBRDUW3, CtD *pgp3*T212A, CtD *pgp4*A37T, and CtD *pgp3*T212A *pgp4*A37T as previously described (47), referred to herein as CtD pBRDUW3, CtD *pgp3*^–^, CtD *pgp4*^–^, and CtD *pgp3*^–^ *pgp4*^–^. The C. trachomatis A2497 *pgp3*-C304T strain was made by ethyl methanesulfonate (EMS) mutagenesis as previously reported (38). The A2497 *pgp3*^–^ strain contains five additional single nucleotide mutations relative to the reference strain (NCBI reference sequence NC_017437 [see Table S1 in the supplemental material]). These single nucleotide polymorphisms (SNPs) do not result in nonsense mutations. All strains were plaque cloned, propagated in McCoy cells, and purified as previously described (48).

10.1128/mBio.01902-20.2 TABLE S1 *De novo* genome sequencing of A2497 *pgp3*^–^. Download Table S1, TIF file, 0.3 MB. Copyright © 2020 Yang et al. 2020 Yang et al. This content is distributed under the terms of the Creative Commons Attribution 4.0 International license.

### Murine model of genital tract infection.

Six- to 8-week-old female C57BL/6 mice were obtained from Taconic Laboratories through a NIAID contract. CRAMP^−/−^ (stock number 017799) mice were purchased from Jackson Laboratories. Mice were injected with 2.5 mg of medroxyprogesterone (Depo-Provera; Pharmacia & Upjohn, NY) subcutaneously at 10 and 3 days prior to chlamydial infection and transcervically infected with 1 × 10^5^ inclusion-forming units (IFU) of Ct in SPG buffer (10 mM phosphate [pH 7.2] containing 0.25 M sucrose and 5 mM l-glutamic acid) (32). The course of infection was monitored by swabbing the vaginal vault with calcium alginate swabs (Puritan Medical, Guilford, ME) at selected intervals, followed by enumeration of recoverable IFU (rIFU) on McCoy cell monolayers as described previously (49). Mouse genital tracts (three mice/group) were harvested at day 60 postinfection (p.i.) for hematoxylin and eosin staining and immunohistochemistry. All procedures were carried out in the animal facility at the NIAID and performed in accordance with the Institutional Animal Care and Use Committee guidelines.

### Macaque model of ocular infection.

Cynomolgus macaques (Macaca fascicularis) were maintained at the Rocky Mountain Laboratories (RML) and cared for under standard practices implemented by the Rocky Mountain Veterinary Branch. Macaques (*n* = 4) were infected by inoculation of the A2497 trachoma strain or the A2497 *pgp3*^–^ strain (2 × 10^6^ IFU) onto the upper and lower conjunctival surfaces of both eyes (50). Clinical evaluation of ocular disease was performed weekly by RML veterinary staff blind to macaque conditions. Ocular clinical disease was scored on the basis of hyperemia and follicle formation on the upper conjunctival surfaces of both eyes. Hyperemia was scored as follows: 0, no hyperemia; 1, mild hyperemia; and 2, severe hyperemia. Subepithelial conjunctival follicles were scored as follows: 0, no follicles; 1, 1 to 3 follicles; 2, 4 to 10 follicles; 3, >10 follicles; and 4, follicles too numerous to count. A composite disease score for each animal was calculated by adding pathology scores found for both eyes. The maximum disease score is 12 (50). Ocular infection was monitored by swabbing the conjunctiva and culturing rIFU on HeLa cell monolayers as previously described (50). Macaques were monitored for shedding and disease for 100 days p.i. All experimental procedures were approved by the RML Animal Care and Use Committee and performed in accordance with the Institutional Animal Care and Use Committee guidelines. The facilities are fully accredited by the American Association for Accreditation of Laboratory Animal Care.

### Inhibition of C. trachomatis *in vivo* and *in vitro* infectivity by the cathelicidin peptide CRAMP.

Six- to 8-week-old female C57BL/6 and CRAMP^−/−^ mice were treated with 2.5 mg of medroxyprogesterone subcutaneously at 10 and 3 days prior to chlamydial infection. Mice were transcervically infected with 1 × 10^5^ IFU of CtD *pgp3*^–^ in SPG buffer. The course of infection was monitored by swabbing the vaginal vault at selected intervals followed by enumeration of rIFU on McCoy cell monolayers as described previously (49).

We performed two *in vitro* assays to identify the mechanism by which Pgp3 inhibited CRAMPs to avoid killing by AMPs. To determine whether Pgp3 associated with the chlamydial outer membrane complex neutralized CRAMP, 1 × 10^5^ elementary bodies (EBs) of CtD, CtD P^−^, CtD pBRDUW3, and CtD *pgp3*^–^ in 100 μl SPG buffer were treated with different concentrations of CRAMP (AnaSpec) at room temperature for 1.5 h. The EB CRAMP suspensions were inoculated onto McCoy cells to assess infectivity and are expressed as rIFU. To determine whether cytosolic secreted Pgp3 neutralized CRAMP, McCoy cells infected with CtD, CtD P^−^, CtD pBRDUW3, and CtD *pgp3*^–^ (multiplicity of infection [MOI] of 0.5) were lysed with osmotic buffer (0.1× phosphate-buffered saline [PBS]) at 44 h p.i. One hundred-microliter samples of the supernatants were incubated with different concentrations of CRAMP at room temperature for 1.5 h. The mixtures were inoculated onto McCoy cells to determine rIFU.

### H&E staining and immunohistochemistry.

Female mouse genital tracts were collected at day 60 postinfection. Female genital tract tissue was fixed in formalin and processed into paraffin wax blocks. Samples were fixed in formalin and processed into paraffin wax blocks. Sections were processed for hematoxylin and eosin (H&E) staining. Immunohistochemical staining was conducted on the Bond RX (Leica Biosystems) platform using established vendor protocols. Briefly, 5-μm-thick sections were deparaffinized and rehydrated. Slides were then incubated with Protein Block X0909 (Dako/Agilent) for 30 min prior to application of primary antibodies (Iba1, Wako catalog no. 019-19741, 1:800; wide-spectrum cytokeratin, Abcam ab9377, 1:100; myeloperoxidase, Abcam ab9535, 1:30) for 1 h. Horseradish peroxidase (HRP) enzyme was conjugated to the primary antibodies by a 60-min incubation with biotinylated horse anti-mouse IgG antibody followed by a 30-min incubation with HRP-streptavidin (Vector Laboratories). Detection with diaminobenzidine (DAB) chromogen and counterstaining with hematoxylin were performed using the Bond Polymer Refine detection kit (Leica Biosystems). Sections were examined by light microscopy using an Olympus BX51 microscope, and photomicrographs were taken using an Olympus DP73 camera.

### Immunofluorescence.

HeLa 229 cells were plated on glass coverslips in 24-well plates and infected with different Ct strains at an MOI of 0.2. The infected cells were fixed with 4% formaldehyde solution (ChemCruz) for 30 min at 40 h p.i. and stained with anti-Pgp3 mouse polyclonal antibody and anti-major outer membrane protein (anti-MOMP) rabbit polyclonal antibody. The secondary antibodies goat anti-mouse conjugated with Alexa Fluor 568 and goat anti-rabbit conjugated with Alexa Fluor 488 (Thermo Fisher) were used at 1:800. Coverslips were further stained with 4′,6′-diamidino-2-phenylindole (DAPI) at 1:1,000 in PBS for 5 min and mounted using ProLong gold. Images were captured on a Nikon Eclipse 80i fluorescence microscope and analyzed using Nikon Elements software.

### Immunoblotting.

McCoy cells were seeded in 24-well plates and infected with different Ct strains at an MOI of 1. Cells were lysed with radioimmunoprecipitation assay buffer at 40 h pi. Samples were subjected to sodium dodecyl sulfate-polyacrylamide gel electrophoresis (SDS-PAGE) and transferred to polyvinylidene difluoride (PVDF) for immunoblotting. The blots were blocked overnight at 4°C in PBS with 0.1% Tween 20 and 5% nonfat dry milk and probed with mouse anti-Pgp3, anti-CT143, anti-CT144, and anti-Hsp60 monoclonal antibodies (MAbs). Horseradish peroxidase-conjugated secondary antibodies were used for detection (Invitrogen).

### Statistical analyses.

GraphPad Prism 7.0 software was used for data analysis. Statistical parameters, including the exact value of *n* (mean ± standard deviation [SD]) and statistical significance, are described in the figure legends.

### Data availability.

Genome sequence data are available in GenBank or the NCBI (accession no. NC_017437) as described previously (17).
