# Supplementary figures and images for: Chlamydia trachomatis Plasmid Gene Protein 3 Is Essential for the Establishment of Persistent Infection and Associated Immunopathology
Source: mBio. 2020 Aug 18;11(4):e01902-20. doi: 10.1128/mBio.01902-20 (PMC7439461; doi:10.1128/mBio.01902-20)

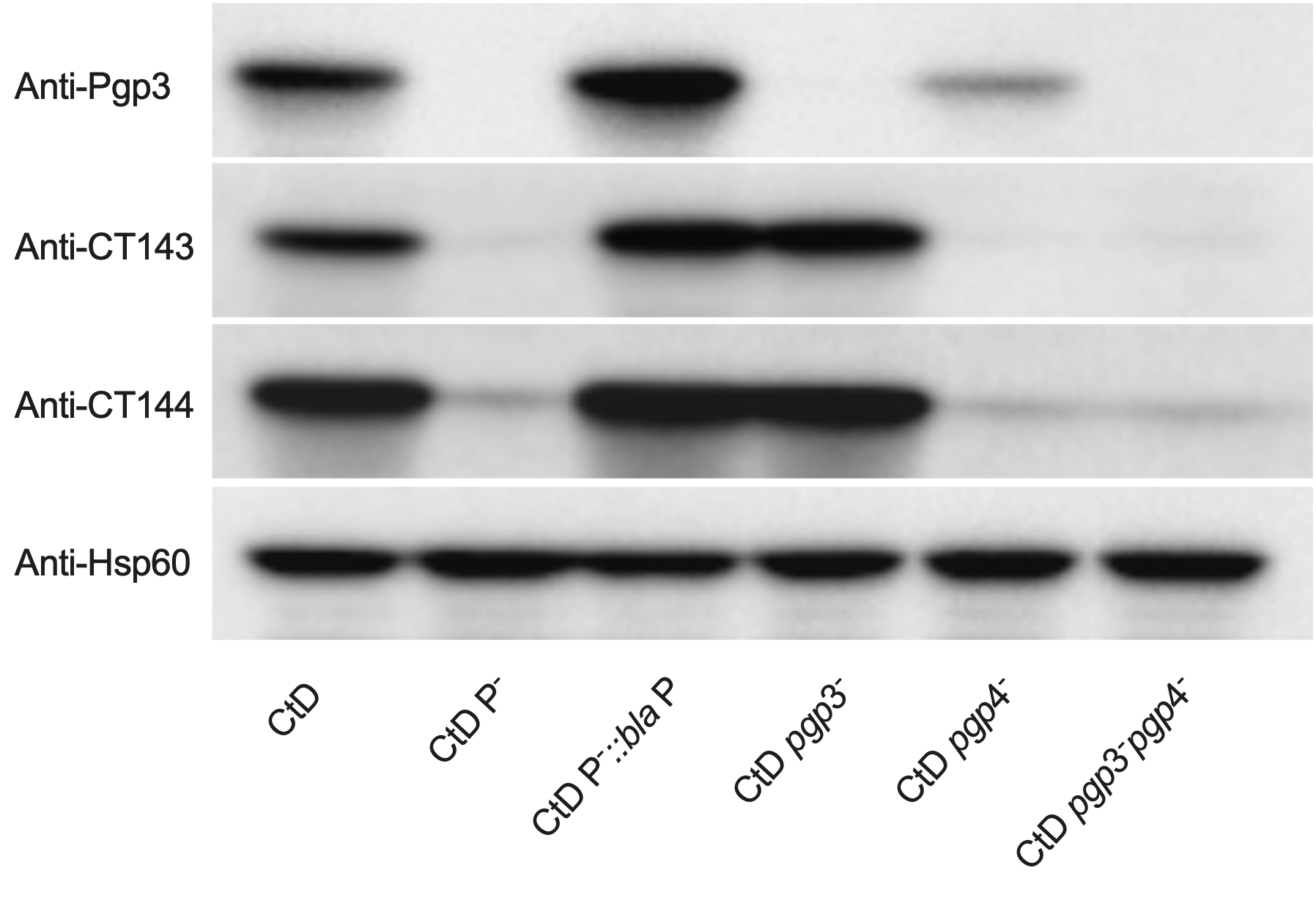

Supplement: FIG S1 [file mBio.01902-20-sf001.tif]

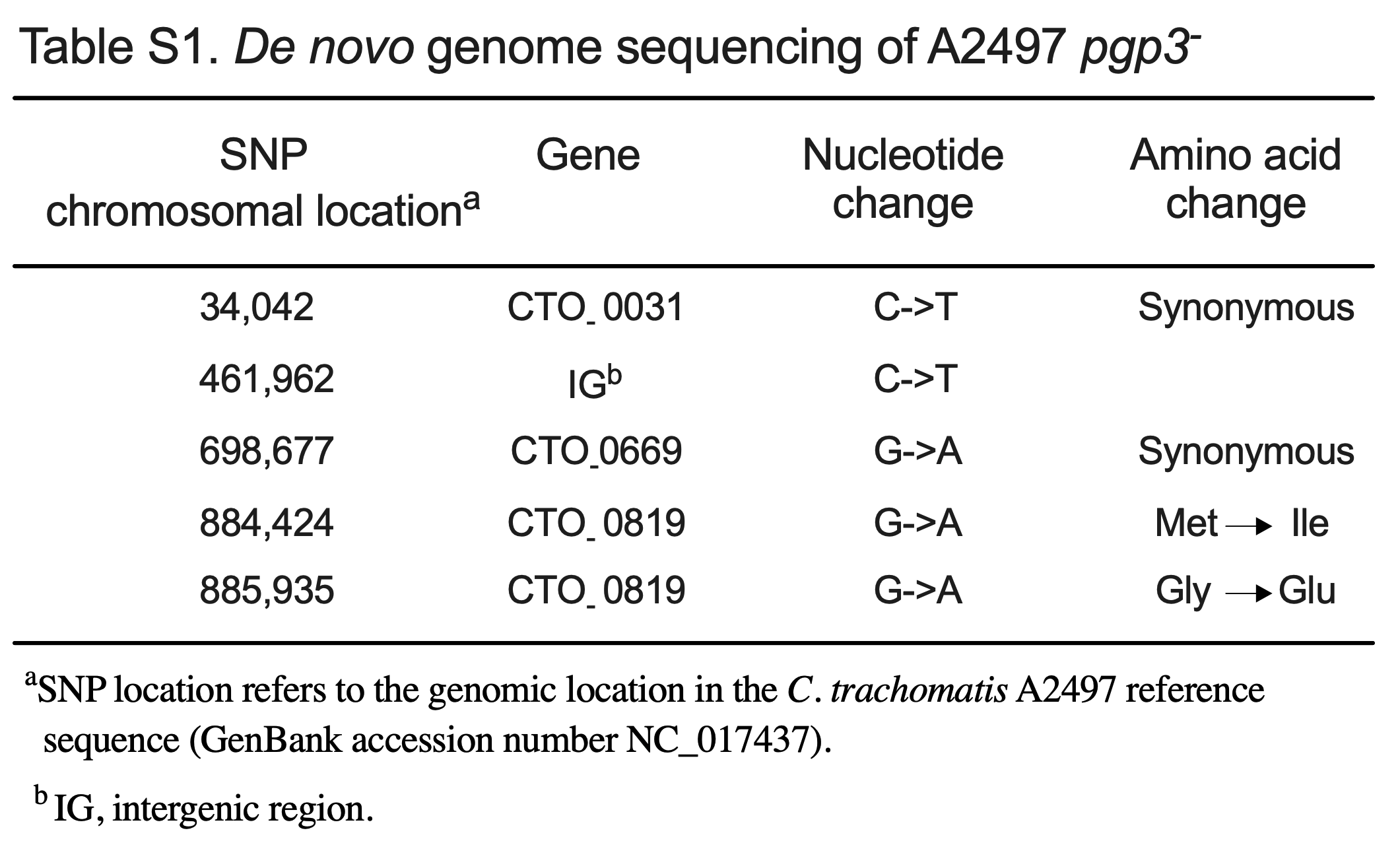

Supplement: TABLE S1 [file mBio.01902-20-st001.tif]
